# A Brief, Individualized Exercise Program at Intensities Below the Ventilatory Threshold Exerts Therapeutic Effects for Depression: A Pilot Study

**DOI:** 10.3389/fnbeh.2021.787688

**Published:** 2021-11-22

**Authors:** Yuri Sakai, Chong Chen, Atsuhito Toyomaki, Naoki Hashimoto, Kan Kitagawa, Takao Inoue, Asumi Sato, Keisuke Makihara, Rie Kameyama, Yumi Wakatsuki, Niki Udo, Ryosuke Shirakawa, Takashi Yokota, Shin Nakagawa, Ichiro Kusumi

**Affiliations:** ^1^Department of Psychiatry, Hokkaido University Graduate School of Medicine, Sapporo, Japan; ^2^Division of Neuropsychiatry, Department of Neuroscience, Yamaguchi University Graduate School of Medicine, Ube, Japan; ^3^Department of Health Sciences, Hokkaido University Graduate School of Health Sciences, Sapporo, Japan; ^4^Department of Cardiovascular Medicine, Faculty of Medicine and Graduate School of Medicine, Hokkaido University, Sapporo, Japan

**Keywords:** depression, exercise, therapeutic, cardiopulmonary exercise testing (CPX), anaerobic threshold (AT)

## Abstract

Due to the fact that existing pharmacological treatments for depression are not ideal, effort has been devoted to the development of complementary, alternative therapies such as physical exercise. The antidepressant effect of exercise is well documented. However, current recommendations and prescriptions of exercise may be too demanding for depressed patients, as some complain about the design of exercise programs and depression is associated with reduced motivation and capacity to exercise. Therefore, appropriately designed, patient-friendly exercise programs may prove critical for the long-term maintenance and therapeutic effects of exercise. In this pilot study, we developed an exercise program based on patients’ individual level of ventilatory threshold (VT), a submaximal index of aerobic capacity measured by Cardiopulmonary Exercise Testing (CPX). Compared to traditional measures, CPX provides more trustable indices of aerobic capacity and more homogenous exercise prescriptions. The main episode of the program consisted of 15–25 min of cycling twice a week at an intensity that approached but never went higher than subjects’ VT (considered low to moderate in intensity). We found that in patients diagnosed with major depressive disorder or persistent depressive disorder (*n* = 8), the program resulted in a significant reduction in depressive symptoms at week 8, which was maintained at week 16. Meanwhile, patients’ social functioning, quality of life, and cognitive functions improved. Although we used a single arm, non-randomized design, our results suggest that even a brief, low to moderate intensity exercise program may exert therapeutic effects for depression and CPX may be a useful tool for exercise prescriptions.

## Introduction

Currently, one of the primary clinical strategies for the treatment of depression is pharmacological therapies. However, merely 60–70% of patients respond to various antidepressant treatments, while 10–30% exhibit treatment-resistant symptoms (Al-Harbi, 2012). The latter experiences substantial social and occupational difficulties, the decline of physical health, and suicidal thoughts, and have increased health care utilization. Consequently, the exploration of non-pharmacological, complementary and alternative therapies has been attracting much attention (Kessler et al., 2001). One such therapy is physical exercise (Cooney et al., 2013; Chen, 2017).

A Cochrane meta-analysis concluded that exercise led to a moderate reduction in depressive symptoms, equivalent to roughly 5 points on the Beck Depression Inventory (BDI) (Cooney et al., 2013). Typically, the exercise used is moderate to vigorous in intensity and at least three times per week. As such, the UK National Institute for Health and Clinical Excellence recommends structured exercise lasting 45 min to an hour, 3 times a week for 10–14 weeks, for the treatment of mild to moderate depression (National Institute for Health and Clinical Excellence, 2016). The Japanese Society of Mood Disorders recommends 3 sessions of exercise at moderate to vigorous intensity for the treatment of major depressive disorder (MDD) (The Japanese Society of Mood Disorders, 2016).

However, these recommended frequencies and intensities of exercise may be inadequate for depressed patients. As reported by previous studies, some patients complain about the design of the exercise programs (Blumenthal et al., 1999, 2007; Oertel-Knöchel et al., 2014). In fact, the widely prescribed frequency and intensity of exercise may be too demanding for these patients. First, depression is associated with reduced motivation to exercise (Roshanaei-Moghaddam et al., 2009; Searle et al., 2011). Second, patients with depression show reduced aerobic capacity (Martinsen et al., 1989; Sener et al., 2016) and walk slower than non-depressed people (Lemke et al., 2000).

Therefore, appropriately designed exercise programs with shorter episodes, fewer sessions and at an individualized, relatively low intensity may prove critical for the long-term maintenance of exercise in patients. But as the therapeutic effect of exercise is generally dose-dependent (Cooney et al., 2013; Chen, 2017), it is of crucial importance to specify the adequate amount of exercise that is friendly and tolerable to patients and still achieves an antidepressant effect.

Cardiopulmonary Exercise Testing (CPX) has been widely used in cardiac rehabilitation for the objective evaluation of cardiorespiratory function (Balady et al., 2010). One measure generated by CPX is the ventilatory threshold (VT), a submaximal index of aerobic capacity (Beaver et al., 1986). As the intensity of exercise increases, the oxygen requirement by the muscle becomes greater. When the oxygen requirement surpasses the oxygen supply, the body has to depend on anaerobic glycolysis for energy output, with lactate as a final metabolic byproduct. This point at which lactate accumulates faster than its rate of use in the blood is known as anaerobic threshold (AT) or lactate anaerobic threshold (LAT). The VO_2_ at the onset of blood lactate accumulation is called VT or ventilatory anaerobic threshold (VAT). VT or VAT is detected using incremental exercise-induced gas exchange parameters, while AT or LAT is detected by direct metabolic measures (e.g., lactic acid). An increase in ventilation is required to eliminate the excess carbon dioxide (CO_2_) produced during the conversion of lactic acid to lactate. This causes the shortness of breath and the feeling of effortful.

Exercise at an intensity below VT is perceived as less demanding. Specifically, in healthy subjects, VT usually occurs at 45–65% of maximal aerobic capacity (VO_2_max) (Davis et al., 1976) and exercise at an intensity below VT is thus considered low to moderate in intensity (Swain and Franklin, 2006).

To our knowledge, to date, no study has evaluated the therapeutic effect of exercise at an intensity below VT—at low to moderate intensity—in depressed patients. Previous studies conducted in depressed patients have generally used exercise at moderate to vigorous intensity, for instance, at 50–85% of heart rate reserve (maximum heart rate minus resting heart rate) (Blumenthal et al., 1999, 2007; Penninx et al., 2002).

Furthermore, the measures employed by previous studies in evaluating the intensity of exercise training in depressed patients, such as heart rate reserve (Blumenthal et al., 1999, 2007; Penninx et al., 2002), has recently been found to be a somewhat unreliable estimate of aerobic capacity (Solheim et al., 2014). In contrast, CPX can provide more trustable indices of aerobic capacity and more homogenous exercise prescriptions (Mann et al., 2013). To date, no study has employed CPX to monitor the intensity of exercise training in depressed patients.

In this pilot study, we aimed to investigate the therapeutic effects of an exercise program developed based on patients’ individual level of aerobic capacity. Specifically, employing CPX, we measured each patient’s VT and asked them to exercise (cycle using an ergometry) twice a week at an intensity below their VT. We hypothesized that exercising twice a week at an intensity below VT reduces depressive symptoms in clinical patients. Furthermore, this may be accompanied by reduced anxiety symptoms and improved sleep quality, social functioning, quality of life, and cognitive functions, etc.

## Materials and Methods

### Subjects

We recruited outpatients throughout the period of February to August 2017 at Hokkaido University Hospital, where the study was approved by the Institutional Review Board. The inclusion criteria were (1) being diagnosed with MDD or persistent depressive disorder (dysthymia) by a senior psychiatrist during clinical interviews according to Diagnostic and Statistical Manual of Mental Disorders (DSM)-5, (2) aged 20–60 years, and (3) being able to provide a written informed consent after being explained the details of the study. The exclusion criteria were (1) severe depression, as indicated by scoring 20 or above on the Hamilton Rating Scale for Depression (HAM-D), (2) strong suicidal thoughts, (3) high manic symptoms, as indicated by scoring 13 or above on the Young Mania Rating Scale (YMRS), (4) active inflammatory disease, (5) malignant tumors, (6) being unable to exercise due to orthopedic diseases, (7) already exercising regularly, (8) other conditions when considered inappropriate by the research director (for instance, body weight greater than the allowance of the ergometry used for exercise, see below).

### Study Design and Procedures

As a pilot study, we used a single arm, non-randomized design. Subjects attended the exercise program while continued their routine pharmacological treatment. The changes in their symptoms from before to after the exercise program were used to assess the therapeutic effect of the exercise program.

The exercise program consisted of two supervised sessions per week for 16 consecutive weeks. Each session lasted 45–55 min and was conducted between 10:00 to 11:30 am. It was initialized by a 10-min stretching exercise and a 5-min warm-up cycling on an ergometry (type 75XL II, 75XL III, or 800, Combi Corporation, Tokyo, Japan). The main episode of exercise was a 15–25 min cycling at an intensity that approached but never went higher than subjects’ VT. Specifically, heart rate was used to monitor the exercise intensity. If subjects’ heart rate exceeded their level of heart rate at VT, the load was reduced by 5–10 watts/min and subjects’ heart rate confirmed again. This process was repeated until subjects’ heart rate decreased to below the level of heart rate at VT. Meanwhile, the intensity of the cycling was further moderated in a way such that subjects’ perceived exertion stayed below 13 (somewhat hard) as evaluated by the Borg Rating of Perceived Exertion Scale. Each session was then finalized by a 5-min cool-down cycling followed by another 10-min stretching exercise.

### Measurements

Before the start of (week 0), amid (week 8), and at the end of (week 16) the exercise program, we evaluated subjects’ symptoms of depression and anxiety, sleep quality, quality of life, and social functions using the following measurements.

*Depressive symptoms*. HAM-D, Clinical Global Impressions-Severity Illness Scale (CGI-S), and BDI-II

*Anxious symptoms*. State-Trait Anxiety Inventory-JYZ (STAI)

*Sleep quality*. Pittsburgh Sleep Quality Index (PSQI)

*Quality of life*. 36-Item Short Form Health Survey^®^ (SF-36v2)

*Social functions*. Social Adaptation Self-evaluation Scale (SASS)

Whereas HAM-D and CGI-S were evaluated by a structured interview, other measures were all self-assessment.

At week 0 and 16, CPX was conducted with an upright electromechanical ergometric bicycle using a ramp protocol (15–25 watts/min) to evaluate VT and VO_2_max. During CPX, respiratory gas analysis was simultaneously performed with a breath-by-breath apparatus (Aeromonitor AE-310S, Minato Medical Science, Osaka, Japan). Finally, the VT was determined by the V-slope method (Beaver et al., 1986). Specifically, VT was defined as the non-linear point of increase in the slope of CO_2_ production versus oxygen uptake during the incremental exercise. Body weight and height were also measured. Finally, the following tests were employed to evaluate cognitive functions.

*Cognitive functions*. The following tests were conducted according to our previous studies (Toyomaki et al., 2008; Shimizu et al., 2013): Continuous Performance Test (CPT), Wisconsin Card Sorting Test (WCST), Stroop Test, Trail Making Test (TMT), Word Fluency Test (WFT), and Verbal Memory Test (VMT).

### Statistical Analysis

The statistical analysis was conducted with IBM SPSS Statistics 26.0. The normality of the data was checked using the Shapiro–Wilk test. Repeated-measures analysis of variance (ANOVA) or the Friedman test (when necessary) was used to assess the effect of the exercise program on depressive and anxious symptoms, sleep quality, quality of life, and social functions, with time as the within-subjects factor (week 0, 8, and 16). When necessary, a paired *t*-test or Wilcoxon signed-rank test was used for the *post hoc* pairwise comparison between different time points. Paired *t*-tests or Wilcoxon signed-rank tests (when necessary) were used to compare cognitive functions, VT, VO_2_max, body weight, and height before and after the program. Effect size (Cohen’s *d*) and *post hoc* power analysis was calculated using G^∗^Power Version 3.1.9.7 (Faul et al., 2007). *P* < 0.05 was considered statistically significant. For analyses with *post hoc* pairwise comparisons, the Bonferroni correction was used to adjust the significance level.

## Results

### Subject Characteristics

Among the 12 initially enrolled patients, four dropped out and 8 (66.7%) were eligible for the final analysis. Among those who dropped out, 2 experienced worsening symptoms of depression because of reasons considered irrelevant to the current exercise program, and 2 moved to another city because of work or family related issues.

The final 8 subjects had a mean age of 42.1 ± 12.7 years. 6 (75%) were females, 3 (37.5%) were married, and 1 (12.5%) being employed. 6 (75%) were diagnosed as MDD while the other 2 (25%) persistent depressive disorder. 5 of the 6 patients (83.3%) with MDD suffered from a recurrent episode.

1 subject (12.5%) had a comorbid social anxiety disorder, 1 obsessive-compulsive disorder, and 1 gender identity disorder. The approximate dose equivalent of antidepressants being administered was 139.1 ± 162.7 mg/d of imipramine. The approximate dose equivalent of anxiolytics being administered was 139.1 ± 162.7 mg/d of diazepam. The approximate dose equivalent of antidepressants and anxiolytics did not change between week 0 and 16 (both *p* > 0.10).

### Intervention Effects

**FIGURE 1:**
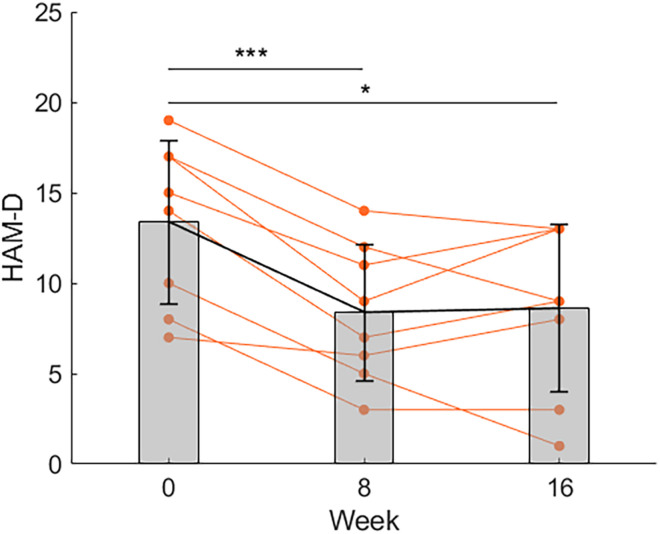
Changes in the score of HAM-D. Compared to before the exercise program (week 0), the mean score of HAM-D significantly decreased at week 8 and maintained at the same level until week 16 (*n* = 8). *p < 0.05, ***p < 0.001. Data is shown as mean ± SD. Orange points represent individual data.

Compared to before the exercise program (week 0), the mean score of HAM-D significantly decreased at week 8 and maintained at the same level until week 16 (Figure 1). Repeated-measures ANOVA revealed a significant effect of time [*F*(2,14) = 17.206; *p* = 0.000]. *Post hoc* comparisons showed that the mean score of HAM-D at week 8 and 16 were lower compared to that at week 0 (*p* = 0.0007, Bonferroni corrected, *d* = 2.42 and *p* = 0.012, Bonferroni corrected, *d* = 1.49, respectively), while the mean score of HAM-D at week 8 was no different from that at week 16. A *post hoc* power analysis for HAM-D scores was conducted. The *post hoc* statistical power for the change at week 8 and 16 compared to week 0 was 0.998 and 0.823, respectively, using a significance level of 0.05/3 and two-sided tests.

Furthermore, the mean score of the Vitality and the Role Emotional (RE) subscale of SF-36v2 increased at week 8 compared to week 0 (see Table 1). Repeated-measures ANOVA revealed a significant effect of time [*F*(2,14) = 5.897, *p* = 0.014 for Vitality; *F*(2,14) = 6.827, *p* = 0.009 for RE] and *post hoc* comparisons showed that the mean score at week 8 was higher compared to that at week 0 (*p* = 0.024, Bonferroni corrected, *d* = 1.30 for Vitality, *p* = 0.027, Bonferroni corrected, *d* = 1.27 for RE, respectively), while the difference between week 0 and week 16 and between week 8 and week 16 were not significant. For the mean score of SASS, the data at week 0 and 8 but not week 16 were normally distributed. We therefore conduced a paired t-test between week 0 and 8 and a Wilcoxon signed-rank test between week 0 and 16 and week 8 and 16. The results showed that the mean score of SASS at week 8 was significant higher compared to that at week 0 (*p* = 0.012, Bonferroni corrected, *d* = 1.47), while the difference between week 0 and week 16 and between week 8 and week 16 were not significant.

**TABLE 1:** Outcome measure before, during, and after the exercise program.

Variables	Week 0	Week 8	Week 16
HAM-D	13.4 ± 4.2	8.4 ± 3.5***	8.6 ± 4.3*
CGI-S	3.5 ± 1.0	3.0 ± 0.5	3.0 ± 0.7
BDI-II	25.0 ± 6.6	20.0 ± 6.3	18.4 ± 6.7
State anxiety, STAI Y1	59.1 ± 6.4	54.3 ± 7.9	55.1 ± 9.2
Trait anxiety, STAI Y2	65.6 ± 7.1	59.9 ± 9.8	56.5 ± 10.9
PSQI	11.1 ± 3.3	10.8 ± 3.1	9.3 ± 4.1
SASS	25.1 ± 6.3	31.1 ± 8.0*	27.4 ± 7.7
SF-36v2: physical functioning	76.3 ± 21.4	79.4 ± 15.6	86.3 ± 20.1
SF-36v2: role physical	71.9 ± 21.9	74.2 ± 18.0	73.5 ± 24.1
SF-36v2: bodily pain	59.9 ± 26.1	65.1 ± 25.1	63.8 ± 27.8
SF-36v2: general health	34.0 ± 21.6	45.4 ± 25.3	44.5 ± 24.2
SF-36v2: vitality	29.7 ± 13.3	45.3 ± 20.0*	41.5 ± 17.9
SF-36v2: social functioning	51.6 ± 10.4	68.8 ± 24.7	73.4 ± 20.8
SF-36v2: role emotional	44.8 ± 15.1	72.9 ± 19.6*	60.4 ± 25.4
SF-36v2: mental health	37.5 ± 13.1	50.0 ± 20.8	49.4 ± 15.2
CPT: error	2.0 ± 1.7	–	1.9 ± 1.5
CPT: response time (milliseconds)	455.0 ± 34.4	–	456.9 ± 46.9
WCST: categories achieved	5.0 ± 0.9	–	5.0 ± 1.2
WCST: perseverative errors of Milner	1.5 ± 2.3	–	1.6 ± 2.1
WCST: perseverative errors of Nelson	2.0 ± 2.8	–	1.9 ± 2.1
Stroop Test: Kanji-controlled	5.4 ± 2.8	–	6.1 ± 2.4
Stroop Test: error	0.5 ± 0.9	–	0.9 ± 1.3
TMT Part A (seconds)	77.0 ± 15.0	–	68.0 ± 10.4*
TMT Part B (seconds)	94.6 ± 19.2	–	83.1 ± 17.1
WFT	26.6 ± 10.5	–	26.0 ± 8.7
VMT: immediate recall	4.6 ± 1.5	–	5.9 ± 1.5
VMT: delayed recall	7.1 ± 2.0	–	8.4 ± 1.9

*p < 0.05, ***p < 0.001 compared to Week 0.

*Data are shown as mean ± SD.*

*HAM-D, Hamilton Depression Rating Scale; BDI-II, Beck Depression Inventory-II; CGI-S, Clinical Global Impressions-Severity Illness Scale; STAI, State-Trait Anxiety Inventory; PSQI, Pittsburgh Sleep Quality Index; SF-36v2, Short Form Health Survey^®^; SASS, Social Adaptation Self-evaluation Scale; CPT, Continuous Performance Test; WCST, Wisconsin Card Sorting Test; TMT, Trail Making Test; WFT, Word Fluency Test; VMT, Verbal Memory Test.*

For the mean score of CGI-S, BDI-II, the trait anxiety subscale of STAI, and the Physical Functioning, General Health, and Mental Health subscales of SF-36v2, repeated-measures ANOVA or the Friedman test indicated a significant effect of time, but *post hoc* comparisons revealed no reliable significant difference among the three time points after Bonferroni correction (data shown in Table 1).

**FIGURE 2:**
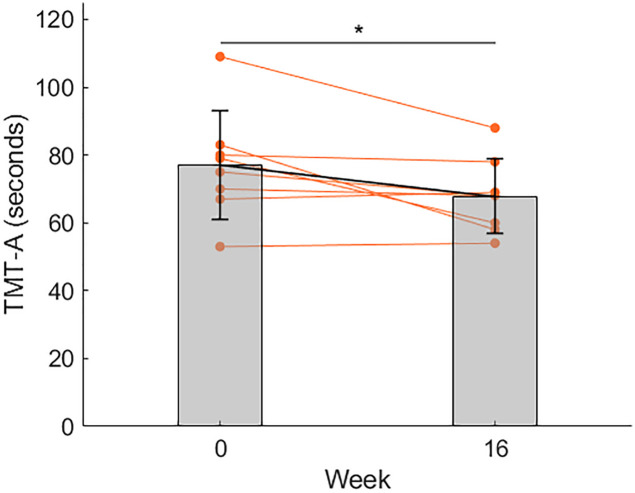
Changes in the reaction time (seconds) of TMT-A. Compared to before the exercise program (week 0), the mean reaction time decreased at week 16. *p < 0.05. Data is shown as mean ± SD. Orange points represent individual data.

For cognitive functions, as shown in Figure 2, the mean time to finish TMT Part A significantly decreased at week 16 compared to week 0, as indicated by a paired *t*-test (*p* = 0.049, *d* = 0.84).

For all other comparisons, no significant difference was detected (data shown in Table 1).

## Discussion

Compared to previous studies which generally prescribed exercise at least 3 times a week and at an intensity of 50–85% of heart rate reserve (Blumenthal et al., 1999, 2007; Penninx et al., 2002), our program is considered at low frequency and low to moderate intensity. Nevertheless, the program resulted in a clinically significant reduction in depressive symptoms at week 8, which was maintained at week 16, as evaluated by the objective measure HAM-D.

Meanwhile, we found that, at week 8, patients’ social functioning and quality of life in terms of vitality and emotional well-being increased. At week 16, patients’ cognitive functions in terms of visual search and motor processing ability improved, as indicated by reduced time to finish relevant tasks. These results suggest that our exercise program may at least partially improve patients’ social and cognitive functioning and enhance their positive emotions such as vitality. These positive outcomes play a pivotal role in patients’ successful rehabilitation and re-adaptation to the social world. Given that most of our patients had recurrent episodes or persistent symptoms, these results are consistent with the observation that exercise prevents the recurrence of depression (Blumenthal et al., 1999).

The dose-dependent effect of exercise in preventing and treating depression has been well-established (Cooney et al., 2013; Chen, 2017). Yet, the introduction and maintenance of high frequency, high intensity exercise in clinical settings is practically difficult. As we have mentioned earlier, depression is associated with both reduced motivation and lower capacity to exercise. Therefore, initiating an exercise program at a patient-friendly, individualized, low frequency, relatively low intensity manner and with brief episodes may be the key toward the maintenance of exercise and the achievement of therapeutic effects.

Notably, among multiple cognitive outcomes, only one test measuring visual search and motor processing ability (i.e., TMT, Part A) showed improvement after the exercise program. Other measures of higher cognitive functions such as cognitive flexibility, inhibitory control, and verbal memory remained unchanged. The underlying explanation of such results is unclear. One possibility is that given the small sample size, our study was underpowered to detect improvement in these higher cognitive functions. Nevertheless, previous studies have generally failed to identify any cognitive effects of exercise in depression (Brondino et al., 2017; Sun et al., 2018). Our study suggests the possibility that exercise at relatively low intensity is perhaps more likely to achieve cognitive-enhancing effects in depressed patients because of a higher rate of adherence (Sun et al., 2018). Future research is required to test such a possibility and clarify the underlying explanation of our findings. The primary limitation of our study is that we used a single arm, non-randomized design and thus could not exclude the potential effect of antidepressants and other factors such as the pure passage of time or repeated testing (i.e., the practice effect on TMT Part A). Our sample size was also relatively small. Future research is necessary to confirm our findings with randomized controlled trials and bigger samples.

In conclusion, our pilot study suggested that even a brief, low to moderate intensity exercise program such as 15–25 min of cycling at an intensity below VT twice a week may have therapeutic effects for patients with depression and that CPX may be a useful tool for exercise prescriptions.

## Data Availability Statement

The data that support the findings of this study are available from the corresponding author upon reasonable request.

## Ethics Statement

The studies involving human participants were reviewed and approved by Hokkaido University Hospital Institutional Review Board. The patients/participants provided their written informed consent to participate in this study.

## Author Contributions

YS, TY, and SN: study concept and design. YS, AT, KK, TI, AS, KM, RS, TY, SN, and IK: acquisition and analysis of clinical data. YS, CC, AT, NH, KK, RK, YW, NU, and SN: interpretation of clinical data. YS, NH, CC, and SN: drafting of the manuscript. All authors critical revision of the manuscript for important intellectual content.

## Conflict of Interest

The authors declare that the research was conducted in the absence of any commercial or financial relationships that could be construed as a potential conflict of interest.

## Publisher’s Note

All claims expressed in this article are solely those of the authors and do not necessarily represent those of their affiliated organizations, or those of the publisher, the editors and the reviewers. Any product that may be evaluated in this article, or claim that may be made by its manufacturer, is not guaranteed or endorsed by the publisher.
